# Vector competence of *Aedes bromeliae* and *Aedes vitattus* mosquito populations from Kenya for chikungunya virus

**DOI:** 10.1371/journal.pntd.0006746

**Published:** 2018-10-15

**Authors:** Francis Mulwa, Joel Lutomiah, Edith Chepkorir, Samwel Okello, Fredrick Eyase, Caroline Tigoi, Michael Kahato, Rosemary Sang

**Affiliations:** 1 Institute of Tropical Medicine and Infectious Diseases (ITROMID), Jomo Kenyatta University of Agriculture and Technology, Nairobi, Kenya; 2 Arbovirus/Viral Hemorrhagic Fever Laboratory, Center for Virus Research, Kenya Medical Research Institute, Nairobi, Kenya; 3 Human health department, International Centre of Insect Physiology and Ecology, Nairobi, Kenya; 4 United State Army Medical Research Directorate- Kenya, Nairobi, Kenya; 5 TRiVE Consortium, College of Health Sciences, Makerere University, Kampala, Uganda; Center for Disease Control and Prevention, UNITED STATES

## Abstract

**Background:**

Kenya has experienced outbreaks of chikungunya in the past years with the most recent outbreak occurring in Mandera in the northern region in May 2016 and in Mombasa in the coastal region from November 2017 to February 2018. Despite the outbreaks in Kenya, studies on vector competence have only been conducted on *Aedes aegypti*. However, the role played by other mosquito species in transmission and maintenance of the virus in endemic areas remains unclear. This study sought to determine the possible role of rural *Aedes bromeliae* and *Aedes vittatus* in the transmission of chikungunya virus, focusing on Kilifi and West Pokot regions of Kenya.

**Methods:**

Four day old female mosquitoes were orally fed on chikungunya virus-infected blood at a dilution of 1:1 of the viral isolate and blood (10^6.4^ plaque-forming units [PFU]/ml) using artificial membrane feeder (Hemotek system) for 45 minutes. The engorged mosquitoes were picked and incubated at 29–30°C ambient temperature and 70–80% humidity in the insectary. At days 5, 7 and 10 post-infection, the mosquitoes were carefully dissected to separate the legs and wings from the body and their proboscis individually inserted in the capillary tube containing minimum essential media (MEM) to collect salivary expectorate. The resultant homogenates and the salivary expectorates were tested by plaque assay to determine virus infection, dissemination and transmission potential of the mosquitoes.

**Results:**

A total of 515 female mosquitoes (311 *Ae*. *bromeliae* and 204 *Ae*. *vittatus*) were exposed to the East/Central/South Africa (ECSA) lineage of chikungunya virus. *Aedes vittatus* showed high susceptibility to the virus ranging between 75–90% and moderate dissemination and transmission rates ranging from 35–50%. *Aedes bromeliae* had moderate susceptibility ranging between 26–40% with moderate dissemination and transmission rates ranging from 27–55%.

**Conclusion:**

This study demonstrates that both *Ae*. *vittatus* and *Ae*. *bromeliae* populations from West Pokot and Kilifi counties in Kenya are competent vectors of chikungunya virus. Based on these results, the two areas are at risk of virus transmission in the event of an outbreak. This study underscores the need to institute vector competence studies for populations of potential vector species as a means of evaluating risk of transmission of the emerging and re-emerging arboviruses in diverse regions of Kenya.

## Author summary

Kenya experienced its first chikungunya outbreak in 2004/2005 along the coastal area, followed by sporadic outbreaks in Mandera in 2016, and subsequently in Mombasa city in late 2017 and early 2018. Despite the rising risk of transmission of the virus in the country based on evidence of outbreaks in Kenya, vector competence studies have only been limited to *Ae*. *aegypti*, while the role played by other *Aedes species* largely remain unknown. This study demonstrated the ability of *Ae*. *bromeliae* and *Ae*. *vittatus* to transmit chikungunya virus under controlled laboratory conditions. Vector competence remains the most important approach in disease risk assessment that provides knowledge to the public health sector in developing vector control guideline.

## Introduction

Chikungunya virus (CHIKV) is vector-borne virus of genus *Alphavirus* and family *Togaviridae* that is principally transmitted from human to humans by *Ae*. *aegypti* and *Ae*. *albopictus*. The first CHIKV outbreak was documented in Makonde village in Tanzania in 1956 [1, 2] and since then, various outbreaks have been experienced in more than 60 countries in Africa, Asia, Europe and America [3, 4]. In Africa high infection was reported in union of Comoros island in the2004- 2005 outbreak [5], Congo in the 1998–2000 outbreaks [6] and Mauritius and Madagascar in 2005 and 2006 respectively [7]. CHIKV is re-emerging in Kenya, after the 2004–2005 outbreaks in Lamu Island. It has caused several outbreaks the northeastern and coastal Kenya from May 2016 and late 2017to early 2018 respectively [8]. In addition, previous studies have reported high seroprevalence rates (59%) of CHIKV infection in Busia District and 24% in Malindi Kenya [9].

Chikungunya virus strains are classified into three distinct genotypes; Asian, West African, and East/Central/South African (ECSA). This virus causes chikungunya fever, an acute febrile illness characterized by severe arthralgia, fever, skin rash, and arthritis-like pain in small peripheral joints that lasts for weeks or months, joint swelling and conjunctivitis [10–12]. Both *Ae*. *aegypti* and *Ae*. *albopictus* have been implicated in the CHIKV transmission cycle in the African region and other parts of the world, based on vector competence studies [13, 14] and virus isolation from infected field collected mosquitoes [15–17]. International travels and global expansion leading to the spread of the two main CHIKV urban mosquito vectors, Ae. *aegypti* and *Ae*. *albopictus*, have enhanced the ability of the virus to spread to new regions where environmental conditions are permissive for viral transmission [18–20]. Extrinsic incubation period (EIP) in mosquitoes infected with CHIKV ranges from 2 to 9 days, with an average of 3 days in the tropics such as East Africa [21]

*Aedes simpsoni* consists of a complex of mosquito species including vectors of important arbovirus diseases such as yellow fever. In Kenya, *Ae*. *bromeliae* is the dominant species of the *Ae*. *simpsoni* complex found in the peridomestic areas, *Ae simponi simpsoni* has never been documented in the country [22]. Studies involving the ecology and vector competence of *Ae*. *vittatus* and *Ae*. *bromeliae* on chikungunya have been conducted in Senegal [23], and on dengue, and yellow fever virus in Kenya [24]. In Rabai, Kenya, *Ae*. *bromeliae* breeds in the domestic and peridomestic areas while *Ae*. *lilii* breeds in the forest [25, 26]. *Aedes bromeliae* preferably feed on human hosts for their blood meal, maintaining the virus in the rural cycle [24] and breed not only on water reservoirs held by plants, including trees holes and plant leaf axils [27, 28], but also in artificial water containers [29, 30].

*Aedes vittatus* is a savannah species that is abundant in rocky areas, prevalent in African forest galleries and is also common in villages near forests. Female *Ae*. *vittatus* have daily and nocturnal activities with a significant crepuscular peak [23, 31]. They bite a wide range of vertebrate hosts, with a strong anthropophilic trend in specific locations [32], and breed mostly on natural habitats mainly in rock pools/holes and tree holes during the rain seasons. In absence of these breeding sites the vector breeds in domestic areas especially in household water-holding containers [23]. The vector has a high susceptibility to infection and dissemination, and most importantly is able to transmit the West Africa lineage of CHIKV [23, 33]. *Aedes vittatus* and *Ae*. *bromeliae* have the potential to expand their distribution and abundance due to their ability to adapt to human dwellings using available breeding habitats, such as domestic containers, in absence of their preferred breeding sites [26, 33, 34].

Determination of the vector competence of mosquito populations is a key parameter in evaluating the risk of CHIKV transmission and spread in Kenya. Despite several outbreaks of CHIKV in Kenya, focus is usually on *Ae*. *aegypti* and no vector competence studies have been conducted to determine the role played by other mosquito species in its transmission and maintenance. We evaluated the competence of *Ae*. *bromeliae* populations from Rabai sub-county in Kilifi County and *Ae*. *vittatus* populations from Kacheliba sub-county in West Pokot County of Kenya as an important factor in assessing the risk of transmission of ECSA lineage of CHIKV in these regions. This would provide the necessary baseline data to inform the public health sector on best vector control practice, and effective preventive and control interventions in case of increased risk of virus transmission.

## Materials and methods

### Study sites

**Fig 1:**
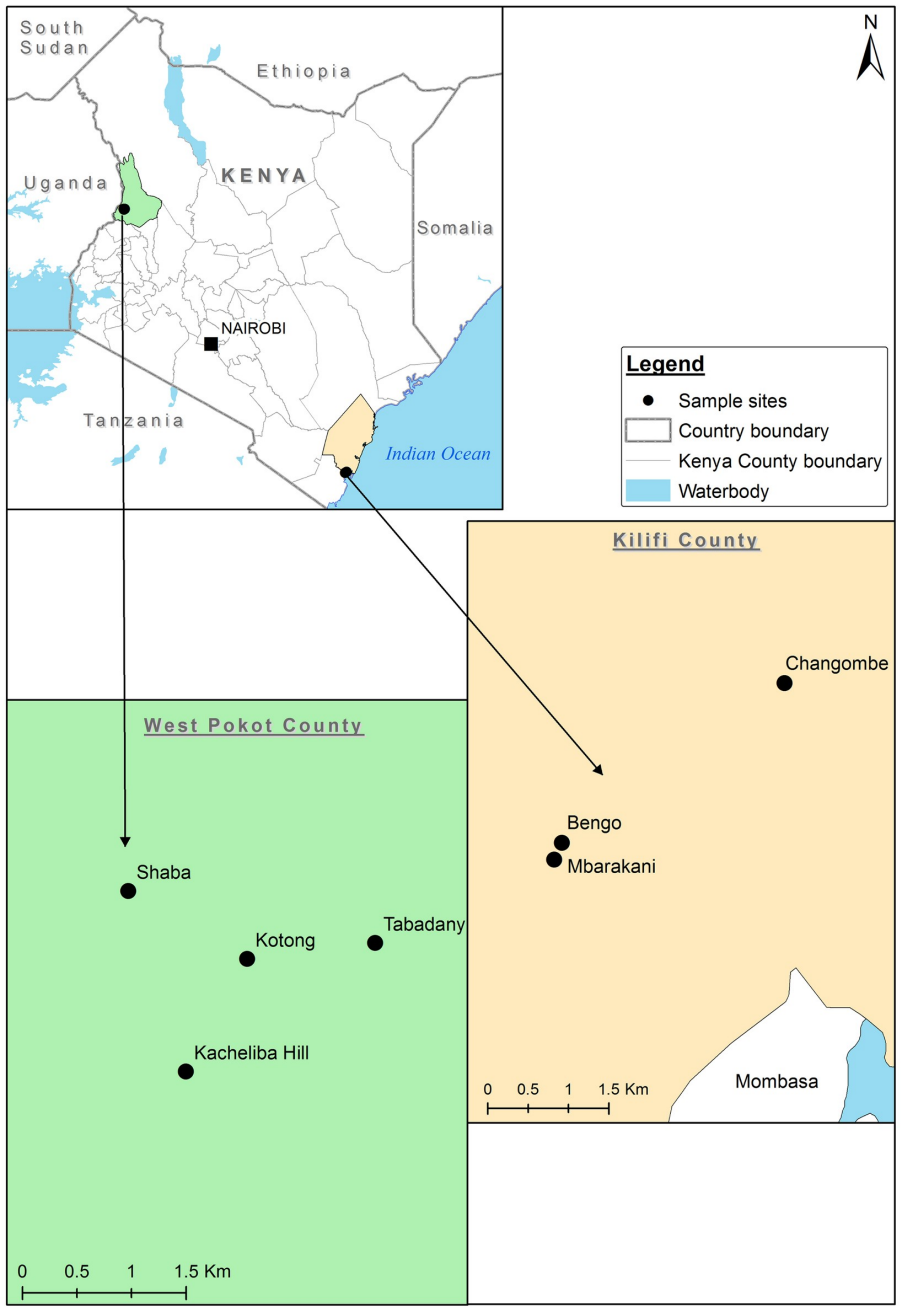
Map of Kenya showing the study sites.

This study was conducted in Rabai sub-county, Kilifi County in the coastal region of Kenya and Kacheliba sub-county in West Pokot County (Fig 1). Kilifi County (latitude 3.63°S, longitude 39.85°E) has a mean annual temperature of 30°C, relative humidity of 82% and receives approximately 88.25 mm of rainfall annually. The county has a bimodal pattern of rainfall with the long rains occurring between April and July, with the highest rainfall occurring in the month of May and short rains in November and December. In Kilifi, the rainfall patterns towards the hinterlands are unreliable due to the influence of the Indian Ocean. The main topographical features include the coastal plains, island plains and Dodori River Plain. The presence of forest areas around the town inhabited by primates and other wildlife species poses a risk of zoonotic disease transmission. Minimum temperatures are always above 20°C, the maximum temperatures reach 30°C to 34°C. The natural vegetation consists of coconut trees, banana plantations and a variety of agricultural crops. Characteristic soil types consist of sandy soil with patches of high loam soil.

West Pokot County lies between latitudes 1.13°N to 2.70°N and longitudes 34.77°E to 35.79°E in the Rift Valley region of Kenya, bordering the Republic of Uganda to the west, Trans-Nzoia County to the south, Elgeyo-Marakwet and Baringo Countiesto the southeast and Turkana County to the north and northeast. It covers an area of 9,169.39 km^2^. West Pokot County has a bimodal rainfall pattern. The long rain season occurs between May and June with mean daily temperature of 32°C, rainfall of approximately 60.25 mm and 82% relative humidity.

### Mosquito collection

*Aedes bromeliae* eggs, larvae and pupae were collected from peridomestic areas in four villages in Rabai sub-county: Mbarakani, Bengo, Changombe and Kibarani (Fig 1). The eggs were collected using ovitraps that consisted of black ovicups lined with oviposition paper and half-filled with water. After obtaining consent from the home/residence owner to sample in their private land, the ovitraps were placed in the peridomestic areas for four days to allow the mosquitoes to lay eggs. Larvae and pupae were collected from natural habitats, mainly rock pools/holes and tree holes, plant axils, especially bananas, and flower axils using larval sampling tools. *Aedes vittatus* larvae and pupae were collected from rock pools/holes and tree holes in peridomestic and forest areas of Kacheliba sub-county. Field collected eggs were briefly dried on a damp cotton wool to induce diapause, and transported to a level 2 (BSL2) insectary at Kenya Medical Research Institute (KEMRI) for colonization.

### Mosquito rearing and identification

To avoid an oviposition from a single female mosquito, several larval collections from the same area were mixed. All collected larvae and pupae were reared to adults in the field laboratory and then transported to the KEMRI insectary for identification. In the insectary, the oviposition papers with eggs were dispensed in water to allow hatching and the emerging larvae were fed on fish fingerlet meal (Tetramin baby) until pupation. The pupae were transferred in small cups containing water to within 4 liter plastic cages with netting material for eventual development to adults. The adults were knocked down at -20°C for 45 seconds and morphologically identified using an identification key [35–38] under a dissecting microscope to select *Ae*. *bromeliae* and *Ae*. *vittatus* for use in the study. The adult mosquitoes were provided with 10% glucose solution on cotton wool and maintained at temperature between 28–32°C, 70–80% relative humidity and 12:12 hour light:dark (L:D) photoperiod. In order to stimulate egg production the mosquitoes were fed on anaesthetized clean laboratory mice placed on top of the cage for 45 minutes. The eggs collected were hatched into F_1_ (first filial generation) and adult mosquitoes were maintained as described.

### Virus and virus amplification

The Lamu001 strain of ECSA lineage CHIKV, isolated from human during the 2004–2005 outbreak in Lamu Island [6], was used for all the infection assays performed in this study. The virus was passaged in confluent monolayers of Vero cells in T-25 cell culture flasks, grown in Minimum Essential Medium (MEM), (Sigma-Aldrich, St. Louis, MO) with Earle’s salts and reduced NaHCO_3_, supplemented with 10% heat inactivated fetal bovine serum **Fetal bovine serum** (or foetal bovine serum) is serum taken from the fetuses of cows. Fetal Bovine Serum (or FBS) is the most widely used serum in the culturing of cells. In some papers the expression **foetal calf serum** is used. (FBS FBS *abbr*. fasting blood sugar FBS Fasting blood sugar. See Fasting glucose.), (Sigma-Aldrich), 2% L-glutamine (Sigma-Aldrich) glutamine (gl`təmēn), organic compound, one of the 20 amino acids commonly found in animal proteins. and 2% antibiotic antimycotic solution containing 10,000 units penicillin, 10 mg streptomycin and 25μg amphotericin B per ml (Sigma-Aldrich, St. Louis, MO). The inoculated monolayer was incubated at 37°C for 1 hour, to allow for virus adsorption and then maintenance medium (MEM, with 2% Fetal Bovine Serum, 2% glutamine, 2% antibiotic/antimycotic) was added and incubated at 37°C. 80% cytopathic effect (CPE) was observed after two days. The **CPE**—Customer Premises Equvirus was harvested, aliquoted in cryovials and stored at -80°C until use [39].

### Virus quantification

Quantification of CHIKV was performed by plaque assay. 10-fold serial dilutions of the amplified CHIKV was carried out and inoculated in 6-well plates containing confluent Vero monolayers as described by Gargan [40]. This was grown in minimum essential medium (MEM), with Earle’s salts and reduced Sodium bicarbonate (NaHCO_3_), supplemented with 10% heat-inactivated Fetal Bovine Serum (FBS), 2% L-glutamine, and 2% antibiotic/antimycotic solution with 10,000 units penicillin, 10 mg streptomycin and 25 μg amphotericin B per ml and incubated at 37°C in 5% CO_2_ overnight. Each well was inoculated with 100 μl of the respective virus dilution, incubated for 1 hour with frequent rocking to allow for adsorption. The infected cells were maintained using 2.5% methylcellulose mixed with 2X maintenance medium (MEM, GIBCO Invitrogen corporation, Carlsbad, California) and incubated at 37°C with 5% CO_2_ for 4 days; then fixed for 1 hour with 10% formalin, stained for 2 hours with 0.5% crystal violet, washed and the plaques counted and calculated to quantify the virus using the following formula [39]:

$$\frac{Number\;of\;plaques}{d\times V}=PFU/ml$$

where d is the dilution factor and V is the volume of diluted virus added to the wells.

### Oral infection of mosquitoes

The wild filial generation (F_0_) and first generation (F_1_) of female *Ae*. *bromeliae* and *Ae*. *vittatus*, respectively, were deprived of glucose for 24 hours before exposure to the infectious blood meal, using an artificial membrane feeding system (Hemotek). The virus/blood mixture was put in membrane feeders covered with freshly prepared mouse skin, and maintained using the hemotek system which employs an electric heating element that maintains the temperature of the blood meal at 37°C. Batches of 50–100 female mosquitoes aged 4–5 days were fed on the virus-blood mixture at a ratio of 1:1 (CHIKV isolate and defibrinated sheep blood) using a Hemotek feeding system for 60 minutes. Only fully engorged mosquitoes were transferred to 4-litre plastic cages (15–30 mosquitoes/cage) with a net on top and maintained with 10% glucose at 28–30°C, relative humidity of 70–80%, and 12:12 hour L: D photoperiod. The non-engorged mosquitoes were destroyed. Mosquito mortality was monitored in the cages by removing and counting dead mosquitoes daily. The experiment was done in three replicates to obtain the sufficient sample size.

### Infection and dissemination rate

On 5, 7 and 10 days post-infection (dpi), a representative sample (at least 30%) of the orally exposed mosquitoes were picked, cold anesthetized and carefully decapitated with the legs/wings and bodies placed into separate 1.5 mL microfuge tubes (Eppendorf). Each mosquito body was placed separately in a well labelled 1.5ml tubes containing 1000 μl of homogenization media (HM), made of MEM, supplemented with 15% FBS, 2% L-glutamine, and 2% antibiotic/antimycotic. Mosquito bodies were homogenized using a mini bead beater (BioSpec Products Inc, Bartlesville, OK 74005 USA) with the aid of a copper bead (BB-caliber airgun shot) and clarified by centrifugation at 12,000 rpm (Eppendorf centrifuge 5417R) for 10 minutes at 4°C. The supernatants were inoculated in Vero cells in 12 well plates, grown in MEM, supplemented with 10% FBS, 2% L-glutamine and 2% antibiotic/antimycotic. One hundred microliters of the appropriate dilutions of the abdominal homogenates was added to each of ten wells of the 12-well plate to infect the cells and the remaining two wells were used for controls, negative control was comprised of male mosquitoes from the study vectors comprising of a pool of 25 mosquitoes. The plates were incubated at 37°C in a 5% CO_2_ incubator with frequent agitation after every 15 minutes for 1 hour to allow for virus adsorption. The infected cell monolayers were then overlaid with 2.5% methylcellulose supplemented with 2% FBS, 2% L-Glutamine and 2% antibiotic/antimycotic and incubated at 37°C in 5% CO_2_. On day 4, plates were fixed for 1 hour with 10% formalin, and stained for 2 hours with 0.5% crystal violet, washed on running tap water, dried overnight and the plaques observed on a light box. The CHIKV positive bodies were used to determine the infection rates. For each positive abdomen, corresponding legs were homogenized and their infection status determined as described above for the abdomens. Plaques were counted and calculated to determine the viral titer. If the virus was detected in the mosquito’s body but not in the legs, the mosquito was considered to have a non-disseminated infection, limited to the midgut. Detection of virus in the body and legs was considered evidence of successful infection and dissemination, respectively [41].

### Test for transmission potential

After exposing the mosquitoes to the infectious blood meal, engorged mosquitoes were picked, placed into new cages, reared under the insectary conditions and maintained with 10% sucrose. On 5, 7 and 10 days dpi, mosquitoes were sucrose-starved and deprived of water for 16 hours, then cold anesthetized for about 40 seconds before the legs and wings from each of them were carefully removed and placed on sticky tape. Individual mosquito proboscises were inserted into a capillary tube containing 10–20 ul HM. Mosquitoes were allowed to expectorate saliva for 30 minutes. Media containing saliva was then expelled into a cryovial containing 200 ul of MEM and stored at -80°C until tested. A volume of 80 μl of the saliva sample was inoculated into each well of a 24-well plate containing confluent Vero cell monolayers. Plates were incubated for 1 hour to allow for adsorption, with frequent agitation. The infected cells were maintained using maintenance media (1 ml per well) and incubated at 37°C with 5% CO_2_. Plates were observed for 7 days and the supernatant of wells showing CPE were harvested and virus quantified by plaque assay as discribed above. Plaques were counted and calculated to quantify the virus.

### Ethical considerations

Scientific and ethical approval to carry out this study was obtained from the KEMRI Scientific Ethical Review Unit (SERU) (KEMRI/SERU/CVR/002/3449). The animal use component was reviewed and approved by KEMRI Animal Care and Use Committee (ACUC) (KEMRI/ACUC/01.05.17). The KEMRI ACUC adheres to national guidelines on the care and use of animals in research and education in Kenya enforced by National Commission for Science, Technology and Innovation (NACOSTI). The Institute has a foreign assurance identification number F16-00211 (A5879-01) from the Office of Laboratory Animal Welfare (OLAW) under the Public Health Service and commits to the International Guiding Principles for Biomedical Research Involving Animals.

### Data and statistical analysis

Three parameters describing vector competence were determined: infection (number of infected mosquito bodies per 100 mosquitoes orally exposed and tested), dissemination (number of mosquitoes with positive legs per 100 mosquitoes infected) and transmission rates (number of mosquitoes with positive saliva per 100 mosquitoes with disseminated infection).

Test of proportions were used to get the infection, transmission and dissemination rates with their 95% confidence interval (CI). Chi-square test with or without Yates’ correction or Fisher’s exact test were used to assess the differences between the two species at each time point and between the three time points for each species. Test of difference between means was done for the titers to determine if there was significant difference in incubation days for each species for the infection and dissemination. Statistical significance was considered for p < 0.05.

## Results

Over 90% of all the larvae and pupae that were collected from plant leaf axils were *Ae*. *bromeliae* while over 70% of larvae and pupae that were collected from rock pools and tree holes were *Ae*. *vittatus*. The mosquito species used in this study and the breeding habitats where they were collected are presented below (Table 1).

**Table 1:** Mosquito species and their preferred breeding habitats.

Mosquito species	Habitat	Breeding sites	Date of collection	Stage
*Ae*. *bromeliae*	Peridomestic	Banana leaf axils	Jul-2017	larvae, pupae
		Arrow root leaf axils	Jul-2017	larvae, pupae
		Flower axils	Jul-2017	larvae, pupae
*Ae*. *vittatus*	Peridomestic	Rock pools/holes	May-2017	Eggs, larvae, pupae
	Forest	Rock pools/holes	May-2017	Eggs, larvae, pupae
		Tree holes	May-2017	Eggs, larvae, pupae

### *Aedes bromeliae* and *Ae*. *vittatus* infection, dissemination and transmission potential

The feeding success rate of the two mosquito species on infected blood meal was high, ranging from 70–80% for *Ae*. *bromeliae* and 40–50% for *Ae*. *vittatus*. The blood meal titres were determined before and immediately after mosquito exposure. The infection rate for Kilifi and West Pokot mosquitoes were measured from a total of 311 *Ae*. *bromeliae* (110 on 5 dpi, 101 on 7 dpi and 100 on 10 dpi) and 204 *Ae*. *vittatus* (69 on 5 dpi, 69 on 7 dpi and 66 on 10 dpi). Both species were susceptible to chikungunya virus infection with average infection rates of 37% and 79% for *Ae*. *bromeliae* and *Ae*. *vittatus*, respectively. *Aedes vittatus* had high midgut infection rate, with no significant difference between the extrinsic incubation periods. The overall dissemination rate was high for *Ae*. *vittatus* with more than 46% of the mosquitoes with midgut infection having a disseminated infection. *Aedes bromeliae* had moderate midgut infection rate on 5 and 7 dpi, but low infection rate on 10 dpi. Overall *Ae*. *bromeliae* showed relatively low dissemination with 34% of those with midgut infection having disseminated infection (Table 2).

**Table 2:** Infection, dissemination and transmission rates of mosquitoes orally infected with CHIKV (infectious blood meal = 10^6.4^ PFU/mL).

		Day 5		Day 7		Day 10	
		n	Infection rate% (95%CI)	n	Infection rate% (95%CI)	n	Infection rate% (95%CI)
Percentage of infection	*Ae*. *bromeliae*	45	40.9 (31.6–50.7)	44	43.6 (33.7–53.8)	26	26.0 (17.7–35.7)
	*Ae*. *vittatus*	56	81.2 (69.9–89.6)	54	78.3 (66.7–87.3)	52	78.8 (67.0–87.9)
	*p* value		**<0.001**		**<0.001**		**<0.001**
Percentage of disemination	*Ae*. *bromeliae*	12	26.7 (14.6–41.9)	16	36.4 (22.4–52.2)	11	42.3 (23.4–63.1)
	*Ae*. *vittatus*	26	46.4 (33.0–60.3)	23	42.6 (29.2–56.8)	26	50.0 (35.8–64.2)
	*p* value		**0.042**		0.531		0.521
Percentage of transmission	*Ae*. *bromeliae*	5	41.7 (15.2–72.3)	5	31.3 (11.0–58.7)	6	54.5 (23.4–83.3)
	*Ae*. *vittatus*	11	42.3 (23.4–63.1)	11	47.8 (26.8–69.4)	9	34.6 (17.2–55.7)
	*p* value		0.97		0.301		0.259

### *Aedes bromeliae* and *Ae*. *vittatus* susceptibility to CHIKV infection, dissemination and transmission

**Fig 2:**
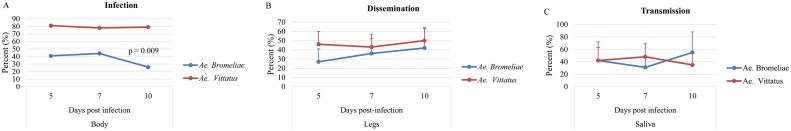
Proportion of Kilifi, *Ae*. *bromeliae* and West Pokot, *Ae*. *vittatus* infected with CHIKV at 5, 7 and 10 days post infection, infection rate (A) and dissemination rate (B) and transmission rate (C).

*Aedes vittatus* was highly susceptible to CHIKV with infection rates of 81%, 78%, and 79% on 5, 7 and 10 dpi respectively compared to *Ae*. *bromeliae* which was moderately susceptible with infection rates of 44%, 41% and 26% on 5, 7 and 10 dpi respectively (Fig 2A). Infection rates for *Ae*. *vittatus* were higher relative to that of *Ae*. *bromeliae*. Statistically significant differences were observed for infection rates 5 dpi between *Ae*. *bromeliae* (40.9%, 95% CI [31.6–50.7%]) and *Ae*. *vittatus* (81.2%, 95% CI [69.9–89.6%]) p < 0.001; 7 dpi between *Ae*. *bromeliae* (43.6%, 95% CI [33.7–53.8%]) and *Ae*. *vittatus* (78.3%, 95% CI [66.7–87.3%]) p < 0.001; and 10 dpi between *Ae*. *bromeliae* (26.0%, 95% CI [17.7–35.7%]) and *Ae*. *vittatus* (78.8%, 95% CI [67.0–87.9%]) p < 0.001 (Table 2). Dissemination rates for *Ae*. *vittatus* were higher relative to those of *Ae*. *bromeliae*. However, statistical significant difference was only observed 5 dpi, *Ae*. *bromeliae* (26.7%, 95% CI [14.6–41.9%]) and *Ae*. *vittatus* (46.4%, 95% CI [33.0–60.3%]) p< 0.042. Viral dissemination was observed as early as 5–7 dpi for both species. The proportion of disseminated infection for *Ae*. *bromeliae* increased significantly with increase in the number of days post infection with higher rate on day 10 (43%). *Aedes bromeliae* had dissemination rate of 26%, 36% and 43% at 5, 7 and 10 dpi (Fig 2B). *Aedes vittatus* had disseminated infection rates of 46%, 43% and 50% at 5,7 and 10 dpi, respectively, but these differences were not statistically significant (chi-square test, p>0.05) (Fig 2B). The overall data shows that 114 out of 277 mosquitos with midgut infection disseminated the virus to the legs, the *Ae*. *vittatus* population from West Pokot County had higher dissemination rate (46%), than the *Ae*. *bromeliae* (34%) population from Kilifi county. Both species were able to transmit the virus as early as 5 dpi. The transmission rate for *Ae*. *bromeliae* was higher on day 10 (55%) compared to other days post infection. *Aedes vittatus* had higher transmission rate on day 7 (48%) which significantly declined on day 10 (35%) post infection (Fig 2C). The overall data for both the Kilifi and West Pokot mosquito population shows that 46 out of 114 (40%) were able to transmit the virus. Although *Ae*. *vittatus* had higher infection and dissemination, there was no significant difference on overall transmission in both vectors (*Ae*. *vittatus* 41% and *Ae*. *bromelia*e 41%).

*Aedes bromeliae* dissemination efficiencies increased with increase in the number of days post infection, *Ae*. *vittatus* had high dissemination efficiencies on 7 dpi (Table 2). Overall transmission rates for *Ae*. *vittatus* was higher (Fig 2) relative to that of *Ae*. *bromeliae* though no statistical significance was observed (chi-square test, p>0.05).

### Chikungunya virus replication dynamics in the analyzed mosquito populations

**Fig 3:**
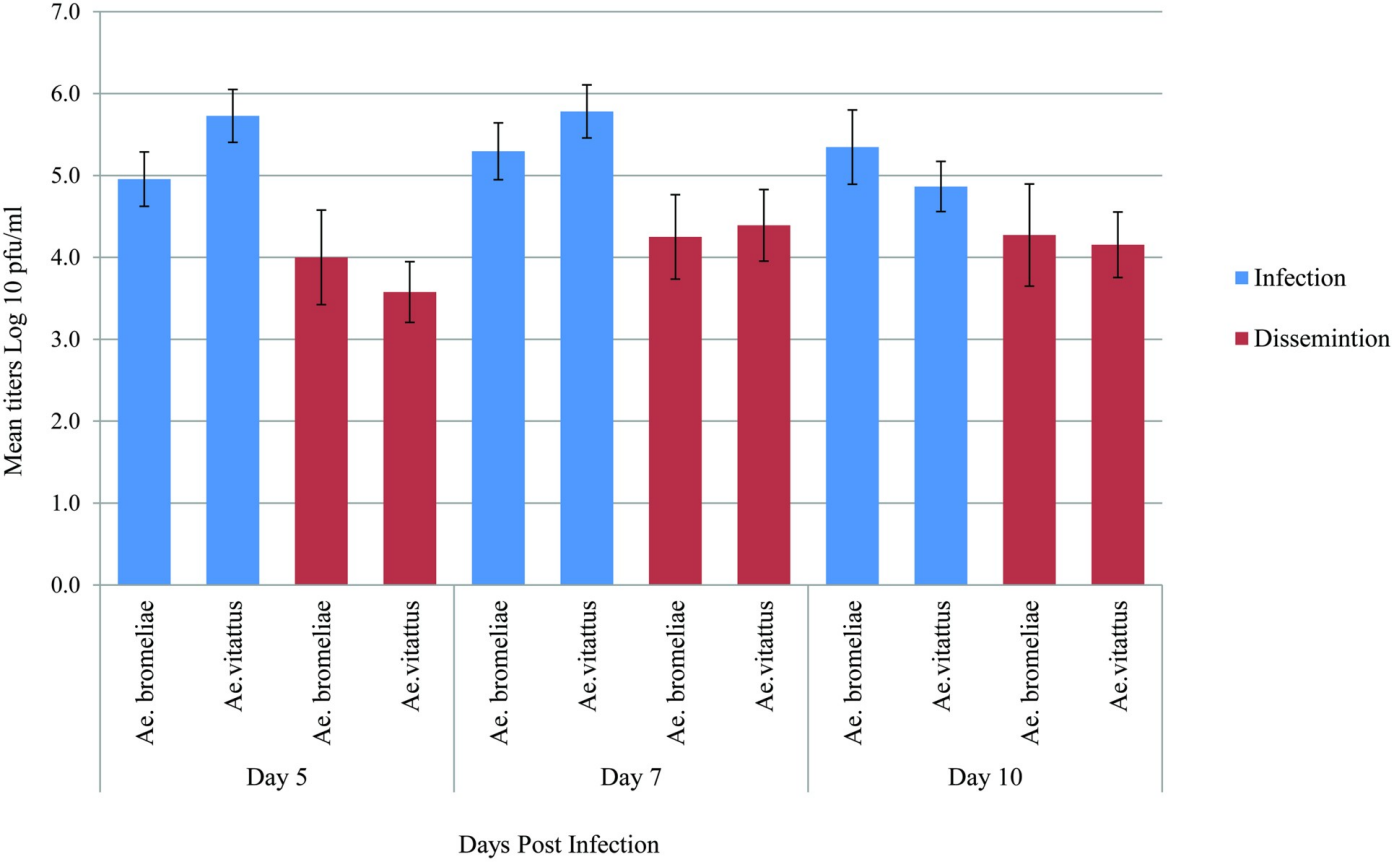
Chikungunya virus replication in *Ae*. *bromeliae* and *Ae*. *vittatus*. Comparisons of CHIKV mean titer in infected *Ae*. *bromeliae* and *Ae*. *vittatus* females was calculated by titration on VERO cells, samples were collected at different days post infection and individually analysed for the presence of CHIKV in body and legs plus wings.

*Aedes bromeliae* and *Ae*. *vittatus* were analysed to assess viral titers in bodies and legs plus wings by titration in Vero cells. *Aedes bromeliae* bodies showed mean viral titers of 5.0 ± 0.33 log_10_ PFU/mL, 5.3 ± 0.34 log_10_ PFU/mL, 5.3 ± 0.45 log_10_ PFU/mL at 5, 7, and 10 days post infection, respectivelyn. The mean CHIKV titers in the bodies increased progressively, reaching a value of 5.3 ± 0.45 log_10_ PFU/mL 10 dpi (Fig 3). The viral presence in the legs was detected as early as 5 dpi with a titer of 4.0 ± 0.58 log_10_ PFU/mL, and titers of 4.3 ± 0.52 log_10_ PFU/mL and 4.3 ± 0.62 log_10_ PFU/mL on day 7 and 10 dpi, respectively.

*Aedes vittatus* bodies showed mean viral titers of 5.7 ± 0.32 log_10_ PFU/mL, 5.8 ± 0.32 log_10_ PFU/mL, 4.9 ± 0.31 log_10_ PFU/mL at 5, 7, and 10 dpi, respectively (Fig 3). The viral presence in the legs was detected as early as 5 dpi with a titer of 3.6 ± 0.37 log_10_ PFU/mL, and titers of 4.4 ± 0.44 log_10_ PFU/mL and 4.2 ± 0.40 log_10_ PFU/mL 7 and 10 dpi respectively. Our results highlighted that among our *Ae*. *bromeliae* and *Ae*. *vittatus* populations, CHIKV was able to infect mosquitoes and replicate over time, disseminating to the wings and legs and reaching the salivary glands. There was no significant difference in infection and dissemination mean titers between the vectors.

In general, viral dissemination only occurred when body titers were ≥ 10^5^ for both strains. *Ae*. *bromeliae* had a midgut infection barrier that was stronger than that of *Ae*. *vittatus*. No difference in leg titers was observed between mosquitoes that did and did not transmit the virus (Table 3). No statistical difference for mean titers for the *Ae*. *bromeliae* and *Ae*. *vittatus* observed for all timepoints (chi-square test, p>0.05).

**Table 3:** Mean body and leg titers for *Ae*. *vittatus* and *Ae*. *bromeliae* exposed to chikungunya virus.

species	Non disseminated	Disseminated ^b^		Mean leg titer ^c^	
	Body titers ^a^	Body titer	Leg titer	No transmission	Transmission
*Ae*. *bromeliae*	10^4.9^	10^5.8^	10^4.1^	10^4^	10^4.3^
*Ae*. *vittatus*	10^5.4^	10^5.9^	10^4^	10^3.9^	10^4.4^

^***a***^ Mean body titer for infected mosquitoes with negative legs (PFU/specimen)

^***b***^ Mean titers for infected mosquitoes with positive legs (PFU/specimen)

^***c***^ Mean leg titers for virus-positive legs with negative saliva (No transmission) and those with positive saliva (Transmission) (PFU/specimen).

## Discussion

This is the first study to determine the ability of *Ae*. *bromeliae* and *Ae*. *vittatus* mosquito populations from Kenya to transmit the ECSA lineage of CHIKV. This study has demonstrated that the two are laboratory competent vectors for ECSA lineage of CHIKV. The recent outbreak of chikungunya in Africa, America, Asia and Europe [18, 42, 43], clearly demonstrates the potential of the disease to spread to new areas and cause massive epidemics. The risk of importation of CHIKV to new areas is due to international and local travels from epidemic areas and exporting infected vectors to new areas where there are susceptible people and competent vectors [14, 44]. The full competence of a vector is not only determined by the ability of the vector to get infected, but also by its ability to transmit the pathogen [45]. In this study we determined the capacity of the vectors to get infected, disseminate and transmit the virus.

The CHIKV titers (10^6.4^ PFU/ml) used to infect mosquitoes in this study, are similar to published viremia levels associated with human infections (often >10^5^ PFU/mL blood) in nature [46]. It has also been shown that a titer of 10^4^ PFU/ml in monkeys was sufficient to infect mosquitoes [41]. Our results show that these two mosquito species are susceptible to infection and have ability to transmit CHIKV (Table 2). Although all mosquito species tested had ingested infectious blood meals, not all mosquitoes were infected and not all that were infected had the virus disseminated. This shows that other factors, such as the midgut escape barrier, affect the replication and dissemination of the virus in a mosquito [47].

The *Ae*. *bromeliae* population had moderate midgut infection which ranged from 26–44% across the different days post infection. Virus infection in the midgut was detected as early as 5 dpi. This is similar to previous studies which showed that the mosquito bodies infection with CHIKV in East Africa ranges from 2–9 days [21]. *Aedes bromeliae* had the highest transmission rate 10 dpi, compared to *Ae*. *vittatus*, which had its highest transmission rate 7 dpi, suggesting *Ae*. *bromeliae* requires more days for the virus to infect the salivary glands and eventually transmit to a susceptible host. Aedes vittatus breeds mostly on rock pools/holes and tree holes as demonstrated by their representing over 70% of the total collected in these habitats. Breeding of Ae. vittatus in rock pools and tree holes has been previously documented [23, 33, 48]. This study showed that the West Pokot population of Ae. vittatus has the potential to transmit CHIKV as has been demonstrated in other studies [23]. Our data showed that Ae. vittatus midgut infection and dissemination rates 5 dpi were relatively high suggesting the presence of weak midgut infection and escape barriers. Our data suggest the West Pokot Ae. vittatus population is efficient in transmitting CHIKV and indicates a potential risk if the virus is introduced in the area.

Our study demonstrated that not all *Ae*. *bromeliae* and *Ae*. *vitattus* are capable of transmitting the CHIKV via capillary feeding; showing that dissemination is dependent on the midgut infection [49]. However, such *in vitro* experiments may not represent the actual amount of virus inoculated in a host during feeding. Despite the two species being exposed to the same virus titers, *Ae*. *vittatus* showed high infection and dissemination rates compared to *Ae*. *bromeliae*. This may be due to other intrinsic factors such as varying strength of midgut infection barrier and midgut escape barrier that individually affect the susceptibility of different mosquito species to infections [50]. It was observed that *Ae*. *vittatus* had a higher midgut infection than *Ae*. *bromeliae*, but there was no significant difference in transmission between the two species regardless of the incubation period. Since this is determined by the ability of the virus to penetrate into the saliva glands and be secreted into the saliva, the data support the notion that the salivary gland barrier is independent of the midgut infection and [51]. For both species, a higher viremia in their infected legs correlated with the ability to transmit the virus by the capillary method. Although this method is not a fully accurate representation of transmission, it does confirm the presence of virus in the salivay and can be used as a model to test for transmission of viruses which have no documented animal models for such experiments. Mosquitoes usually secrete less virus into a capillary tube than when feeding on an animal [52] and transmission rates are often lower when they are determined by collection of saliva as compared to allowing the mosquito to feed naturally on a susceptible animal [53]. Therefore, failure to detect CHIKV in the saliva collected in a capillary tube does not necessarily mean that the mosquito would not have transmitted the virus by bite if it fed on a susceptible human. In this case, our transmission rates should be considered as minimum transmission rates. Additionally, although *Ae*. *vittatus* and *Ae*. *bromeliae* from Kenya are efficient laboratory vectors, their potential role in CHIKV transmission depends on other factors in relation to mosquito ecology such as densities, survival, longevity, anthropophily and duration of gonotrophic cycles, which have been shown to interfere with transmission and maintenance of CHIKV.

## Conclusion

This study demonstrated that *Ae*. *vittatus* and *Ae*. *bromeliae* populations in Kenya are laboratory competent vectors of ECSA lineage CHIKV and it indicates the potential for CHIKV transmission to occur in these locations should the virus find its way there through travel or introduction via a sylvatic host. It is therefore recommended that the public health authorities should continually monitor and carry out surveillance of the CHIKV and virus genotypes circulating within particular regions as well as identify vectors mediating these transmissions to prevent their adverse effects before an outbreak.
